# Engine remaining useful life prediction model based on R-Vine copula with multi-sensor data

**DOI:** 10.1016/j.heliyon.2023.e17118

**Published:** 2023-06-12

**Authors:** Sujuan Liu, Han Jiang

**Affiliations:** College of Artificial Intelligence, Tianjin University of Science & Technology, Tianjin, 300457, China

**Keywords:** Nonlinear Wiener process, R-Vine copula, Remaining useful life, Degradation process, Multi-sensor degradation signals

## Abstract

Aeroengine is a highly complex and precise mechanical system. As the heart of an aircraft, it has a crucial impact on the overall life of the aircraft. Engine degradation process is caused by multiple factors, so multi-sensor signals are used for condition monitoring and prognostics of engine performance degradation. Compared with the single sensor signal, the multi-sensor signals can more comprehensively contain the degradation information of the engine and achieve higher prediction accuracy of the remaining useful life (RUL). Therefore, a new method for predicting the RUL of an engine based on R-Vine Copula under multi-sensor data is proposed. Firstly, aiming at the phenomenon that the engine performance parameters change over time, and the performance degradation presents nonlinear characteristics, the nonlinear Wiener process is used to model the degradation process of a single degradation signal. Secondly, the model parameters are estimated in the offline stage to integrate the historical data to obtain the offline parameters of the model. In the online stage, when the real-time data is obtained, the Bayesian method is used to update the model parameters. Then, the R-Vine Copula is used to model the correlation between multi-sensor degradation signals to realize online prediction of the remaining useful life of the engine. Finally, the C-MAPSS dataset is selected to verify the effectiveness of the proposed method. The experimental results show that the proposed method can effectively improve prediction accuracy.

## 1 Introduction

As the heart of aircraft, the engine operation state plays a decisive role in aircraft safety. The operating environment of an aeroengine is extremely harsh, often accompanied by high temperature and high pressure, which poses a great challenge to the timeliness of engine status detection and maintenance. With the application of condition monitoring technology, it is becoming more common to use sensors to monitor the real-time status of equipment. Prognostic and health management (PHM) technologies can well monitor the health status of equipment, among which remaining useful life (RUL) prediction, as the core of PHM, is widely concerned by researchers [1,2].

The predictive degradation model uses the degradation signals to predict the RUL of the system. The degradation signal can be derived from the raw sensor signal, sometimes involving some signal conversion. Traditionally, the research on RUL prediction methods can be roughly divided into two categories: model-based methods and data-driven methods. Model-based methods require sufficient knowledge of the failure mechanisms of devices and rely heavily on physical principles and engineering experience [2]. However, detailed physical knowledge of the degradation process of the underlying components is difficult to obtain. In recent years, with the increasing complexity of industrial machines and rapid development of state detection technology, data-driven methods have been popularly used in predictive maintenance problems. One of the popular data-driven methods is stochastic process-based approaches. The degradation process of equipment has uncertainty due to failure mechanism and operating environment, which can be effectively captured by stochastic process. Among them, the Wiener process is widely used because of its good mathematical characteristics, which are suitable for describing both monotonic and non-monotonic degradation processes [1]. The Wiener process has a good performance in modeling a single degradation signal process. However, in actual work, many factors affect the engine degradation process, and the correlation between the factors is unknown. This paper uses R-Vine Copula to build the correlation between degradation signals.

The research of RUL prediction methods has made many results. However, most traditional methods assume that signals are independent of each other or RUL prediction based on a single degradation signal [3,4]. However, in the actual situation, signals are often interrelated. When a single degradation signal is used to describe the degradation process of equipment, the RUL can be predicted accurately only when the degradation signal has a strong correlation with the degradation process. Therefore, the accuracy of the RUL prediction based on a single degradation signal is generally unsatisfactory [5], and it is appropriate to model the correlation between multi-sensor degradation signals to improve the accuracy of RUL prediction. The research of RUL prediction based on the Wiener process and multi-sensor data is a hot topic. Si et al. [6] proposed a more widely used nonlinear Wiener process model for RUL prediction. The location parameter of the measurement error that will occur during the actual degradation process, Si et al. [7] used the maximum likelihood estimation method to solve this problem. Liu et al. [8] used an optimal R-Vine Copula information fusion method for failure probability analysis, considering the correlation of failure modes of multiple monitoring points of the main girder of long-span bridges in service. In recent years, RUL prediction based on deep learning has also been extensively studied by scholars. Zhang et al. [9] used KF-EM-RTS to predict the RUL of the battery under the condition of unlabeled small sample data. Zhang et al. [10] proposed a neural network composed of 1-DCNN and BiGRU to predict RUL, which integrated the spatial and temporal features in the measurement data in parallel. Zhang et al. [11] used the modified Transformer-based IMDSSN to predict RUL, which solved the significant limitation of the convolution size of CNN and LSTM when processing temporal data. Zhang et al. [12] used a variational auto-encoder-long–short-term memory network-local weighted deep sub-domain adaptation network (VLSTM-LWSAN) to predict RUL.

The main contribution of the proposed model is the first introduction of R-Vine Copula to model multi-feature non-single correlation in aeroengine RUL prediction for the first time. R-Vine Copula can capture various tail dependencies in a single model, which makes it more accurate when modeling complex multi-feature dependencies. R-Vine Copula is suitable for modeling such scenarios where the correlation is unknown. R-Vine Copula uses the pair Copula function to decompose the multivariate joint distribution, which provides the possibility to model multiple correlations between multiple features. R-Vine Copula has more choices of decomposing dependence structure than d-Vine Copula and C-Vine Copula [13].

In this paper, several degradation signals are modeled separately by the nonlinear Wiener process; in the offline stage, historical data is integrated to obtain offline parameters; in the online phase of parameter estimation, the online parameters of the model are obtained by combining real-time data and offline parameters with Bayesian rule; use the marginal distribution of R-Vine Copula joint degradation features to obtain the joint distribution of degradation signals, and realize the prediction of RUL based on simulation; finally, a simulated turbofan engine dataset is used to verify the effectiveness of the proposed method.

The remainder of this paper is organized as follows. Section 2 introduces the degradation model based on the nonlinear Wiener process, estimates the parameters of the model and obtains the RUL prediction of a single degradation signal. Section 3 uses R-Vine copula to model the correlation between multi-sensor degradation signals. Section 4 carries out RUL prediction with multi-sensor data. Finally, section 5 verifies the effectiveness of the proposed method through experiments.

## 2 Degradation model based on nonlinear Wiener process

### 2.1 Nonlinear Wiener process

Wiener process has good mathematical properties, which can describe both monotone and non-monotone degradation processes. Since the engine performance degradation is nonlinear, the nonlinear Wiener process is used to model the engine performance degradation process, expressed as follows. Let X(t) denote the degradation at time t:

(1) $$X\left(t\right)=X\left(t_{0}\right)+\alpha \int_{0}^{t}\mu \left(\tau ,\beta \right)d\tau +\sigma_{b}B\left(t\right)$$

where $X\left(t_{0}\right)$ represents the initial state; α represents the drift coefficient, reflecting the degradation rate and determining the degradation path; $\sigma_{b}$ is the diffusion coefficient; μ(t,β) is a nonlinear function of time t, which is used to describe the nonlinear characteristics of engine performance degradation, and if μ(t,β)=μ, then Eq. (1) becomes the conventional linear model; B(t) is the standard Brownian motion, which is used to describe the random fluctuations. Without loss of generality, we suppose $X\left(t_{0}\right)=0$. In order to reflect the nonlinearity of the model, this paper sets $\mu \left(t,\beta \right)=\beta t^{\beta -1}$. Considering that the engine is affected by internal and external factors, the degradation process of the engine has great randomness, so this paper takes the drift coefficient α as a random parameter and obeys the normal distribution $\alpha ∼N\left(\mu_{a},\sigma_{a}^{2}\right)$. The nonlinear Wiener process has been widely used in performance degradation modeling of complex systems such as aeroengines, bearings and batteries [14,15].

### 2.2 RUL prediction method of single degradation signal

According to the concept of first hitting time (FHT), the RUL of the equipment is expressed as the time required from the degradation state $X\left(t_{k}\right)$ to the first reaching the failure threshold F. F can be obtained from Eq. (2) [16].

$$L_{k}=\inf\left\{l_{k}:X\left(t_{k}+l_{k}\right)\geq F|X\left(t_{k}\right)<F\right\}$$

(2) $$F=\min\left(x_{1,N_{1}},\cdots ,x_{i,N_{i}}\right)$$

where $L_{k}$ is the RUL of the system at the current time $t_{k}$, $x_{i,N_{i}}$ is the performance degradation of i-th engine at the failure time t.

According to literature [17], if unknown parameter are fixed, the PDF of RUL can be formulated as

(3) $$f_{L_{k}}\left(l_{k}\right)≅\frac{1}{\sqrt{2\pi l_{k}^{2}\left(\sigma_{a}^{2}\eta {\left(l_{k}\right)}^{2}+\sigma_{b}^{2}l_{k}\right)}}\left(\left(F-x_{k}-\eta \left(l_{k}\right)-\beta l_{k}{\left(l_{k}+t_{k}\right)}^{\beta -1}\right)\times \frac{\sigma_{a}^{2}\eta \left(l_{k}\right)\left(F-x_{k}\right)+\mu_{a}\sigma_{b}^{2}l_{k}}{\sigma_{a}^{2}\eta {\left(l_{k}\right)}^{2}+\sigma_{b}^{2}l_{k}}\right)\exp\left[-\frac{{\left(F-x_{k}-\mu_{a}\eta \left(l_{k}\right)\right)}^{2}}{2{(\sigma_{a}^{2}\eta \left(l_{k}\right)}^{2}+\sigma_{b}^{2}l_{k})}\right]$$

where $\eta \left(l_{k}\right)={\left(l_{k}+t_{k}\right)}^{\beta }-t_{k}^{\beta }$, $x_{k}$ is the performance degradation of the engine at time t-th.

The engine RUL can be depicted in Eq. (4) by calculating the expectation from Eq. (3).

(4) $$E\left(l_{k}\right)=\int_{0}^{t}l_{k}f\left(l_{k}\right)dl_{k}$$

### 2.3 Offline parameter estimation

The offline parameters are estimated from historical data on engine performance degradation. Assuming that there are N engines, the degradation data of the i-th engine is, $x_{n,1},x_{n,2},\ldots ,x_{n,m_{n}}$ respectively obtained at time $t_{n,1},t_{n,2},\ldots ,t_{n,m_{n}}$. Therefore, the degradation path of n*-*th item at j-th time point $t_{n}^{j}$ is, from Eq. (1), given by

(5) $$X_{n}\left(t_{n,j}\right)=\alpha_{0}t^{\beta }+\sigma_{b}\left(t_{n,j}\right)$$

$X_{n}={\left(x_{n}\left(t_{n,1}\right),x_{n}\left(t_{n,2}\right),\ldots ,x_{n}\left(t_{n,m_{n}}\right)\right)}^{T}$ represents a collection of performance degradation data for all engines, $T_{n}={\left(T_{n,1},T_{n,2},\ldots ,T_{n,m_{n}}\right)}^{T},T_{n,j}=t_{n,j}^{\beta }$. According to Eq. (5) and the independent increment properties of B(t), it can be known that $X_{n}$ obeys multidimensional normal distribution whose mean and covariance are depicted in Eqs. (6), (7) below:

(6) $$\tilde{\mu }=\mu_{a}T_{n},\Sigma_{n}=\Omega_{n}+\sigma_{a}^{2}T_{n}T_{n}^{T}$$

where

(7) $$Q_{n}=\left[\begin{array}{cccc} t_{n,1} & t_{n,1} & \cdots & t_{n,1}\\ t_{n,1} & t_{n,2} & \cdots & t_{n,2}\\ \vdots & \vdots & \ddots & \vdots \\ t_{n,1} & t_{n,2} & \cdots & t_{n,m_{n}} \end{array}\right],\Omega_{n}=\sigma_{b}^{2}Q_{n}$$

Since devices are independent, the log-likelihood function of parameter $\theta ={\left(\mu_{a},\sigma_{a}^{2},\sigma_{b}^{2},\beta \right)}^{T}$ under X can be depicted in Eqs. (8), (9), (10) [18] below:

$$l\left(\mu_{a},\sigma_{a}^{2},\sigma_{b}^{2},\beta |X\right)=-\frac{\ln\left(2\pi \right)}{2}\sum_{n=1}^{N}m_{n}-\frac{1}{2}\sum_{n=1}^{N}\ln\left|\Sigma_{n}\right|$$

(8) $$-\frac{1}{2}\sum_{n=1}^{N}{\left(X_{n}-\mu_{a}T_{n}\right)}^{\prime }\Sigma_{n}^{-1}\left(X_{n}-\mu_{a}T_{n}\right)$$

where

(9) $$\left|\Sigma_{n}\right|=\left|\Omega_{n}\right|\left(1+\sigma_{a}^{2}T_{n}^{\prime }\Omega_{n}^{-1}T_{n}\right)$$

(10) $$\Sigma_{n}^{-1}=\Omega_{n}^{-1}-\frac{\sigma_{a}^{2}\Omega_{n}^{-1}T_{n}^{\prime }\Omega_{n}^{-1}}{1+\sigma_{a}^{2}T_{n}^{\prime }\Omega_{n}^{-1}T_{n}}$$

Eq. (8) is complicated, and it is difficult to obtain an analytical solution. Therefore, assuming that $\sigma_{a}^{2}$, $\sigma_{b}^{2}$ and β are known, let the first derivative of Eq. (8) equal to 0, and the maximum likelihood estimation result of $\mu_{a}$ is

(11) $${\hat{\mu }}_{a}=\frac{\sum_{n=1}^{N}T_{n}^{\prime }\Sigma_{n}^{-1}X_{n}}{\sum_{n=1}^{N}T_{n}^{\prime }\Sigma_{n}^{-1}T_{n}}$$

Then, the profile likelihood function of $\sigma_{a}^{2}$, $\sigma_{b}^{2}$ and β with respect to the maximum likelihood estimate of $\mu_{a}$ is depicted in Eq. (12) [18] below:

$$l\left(\sigma_{a},\sigma_{b},\beta |X,{\hat{\mu }}_{a}\right)=-\frac{1}{2}\ln\left(2\pi \right)\sum_{n=1}^{N}m_{n}-\frac{1}{2}\sum_{n=1}^{N}\ln\left|\Sigma_{n}\right|-$$

$$\frac{1}{2}\{\sum_{n=1}^{N}X_{n}^{\prime }\Omega_{n}^{-1}X_{n}-2\frac{\sum_{n=1}^{N}T_{n}^{\prime }\Sigma_{n}^{-1}X_{n}}{\sum_{n=1}^{N}T_{n}^{\prime }\Sigma_{n}^{-1}T_{n}}\sum_{n=1}^{N}T_{n}^{\prime }\Sigma_{n}^{-1}X_{n}+$$

(12) $${\left(\frac{\sum_{n=1}^{N}T_{n}^{\prime }\Sigma_{n}^{-1}X_{n}}{\sum_{n=1}^{N}T_{n}^{\prime }\Sigma_{n}^{-1}T_{n}}\right)}^{2}\sum_{n=1}^{N}T_{n}^{\prime }\Sigma_{n}^{-1}T_{n}\}$$

The maximum likelihood estimates of $\sigma_{a}^{2}$, $\sigma_{b}^{2}$ and β are obtained by fminsearch function of MATLAB, and substituted into Eq. (11) to obtain the maximum likelihood estimates of $\mu_{a}$.

### 2.4 Online parameter estimation

In the online phase, assume that when the engine runs to the time $t_{k}$, the observed data $X_{1:k}=\left(x_{1},x_{2},\cdots ,x_{k}\right)$ can be obtained, then its increment is $\Delta X_{1:k}=\left(x_{1},x_{2}-x_{1},\cdots ,x_{k}-x_{k-1}\right)$. After the offline parameters are obtained, for the engine in work, the real-time data is obtained through the sensors, and the posteriori parameters of the model, that is, the online parameters of the model, can be obtained by using the Bayesian chain rule.

According to Bayesian chain rule, when offline parameters $\theta =\left(\mu_{a},\sigma_{a}^{2},\sigma_{b}^{2}\right)$ and real-time data $X_{1:k}$ are known, the posteriori estimation of parameters can be obtained in Eq. (13) [19] below:

(13) $$\left\{ \begin{array}{l} \mu_{k}=\frac{\mu_{a}\sigma_{b}^{2}+\sigma_{a}^{2}x_{k}}{t_{k}\sigma_{a}^{2}+\sigma_{b}^{2}}\\ \sigma_{k}^{2}=\frac{\sigma_{b}^{2}\sigma_{a}^{2}}{t_{k}\sigma_{a}^{2}+\sigma_{b}^{2}} \end{array}\right.$$

## 3 Construction of correlation between multi-sensor degradation signals

Assuming that the correlation between signals is known, traditional methods can easily obtain the correlation between them using the correlation matrix. However, in practice, especially in the engine operating environment, the correlation between multi-sensor degradation signals is often unknown. In this case, traditional methods are difficult to apply, and Copula theory can solve this problem. Various Copula functions, such as Gauss Copula, Clayton Copula, Gumbel Copula and Frank Copula, are suitable for describing sequences with specific tail distribution [20]. R-Vine Copula can use different Copula functions to describe the relationship between different signals, which is suitable for situations where there are multi-sensor degradation signals and the correlation between signals is unknown. Therefore, R-Vine Copula is used to describe the relationship between degradation signals in this paper.

### 3.1 Copula theory

In 1959, Skalr introduced the Copula theory to construct multivariate distribution. Copula theory decomposes multivariate distribution into one Copula function and multiple marginal distributions. The properties of variables are described by their marginal distributions, and the correlation between variables is determined by Copula function [21].

Let $X=\left(X_{1},X_{2},\cdots ,X_{d}\right)$ be a d-dimensional random vector, the joint distribution function of random variables $X_{1},X_{2},\cdots ,X_{d}$ is $F\left(x_{1},x_{2},\cdots ,x_{d}\right)$, and their marginal distribution functions are $F_{1}\left(x_{1}\right),F_{2}\left(x_{2}\right),...,F_{d}\left(x_{d}\right)$, respectively. Then, there is a Copula function C, which satisfies

(14) $$F\left(x_{1},x_{2},...,x_{d}\right)=C\left(F_{1}\left(x_{1}\right),F_{2}\left(x_{2}\right),...,F_{d}\left(x_{d}\right)\right)$$

The form of the joint probability density function of Eq. (14) can be depicted in Eq. (15) below:

(15) $$f\left(x_{1},...,x_{d}\right)=c\left(F_{1}\left(x_{1}\right),...,F_{d}\left(x_{d}\right)\right)\prod_{i=1}^{d}f_{i}\left(x_{i}\right)$$

where c represents the density function of Copula; $f_{i}\left(x_{i}\right)$ is the marginal density function of the variable $X_{i}$.

### 3.2 Correlation between multi-sensor signals in R-Vine copula model

**Fig. 1:**
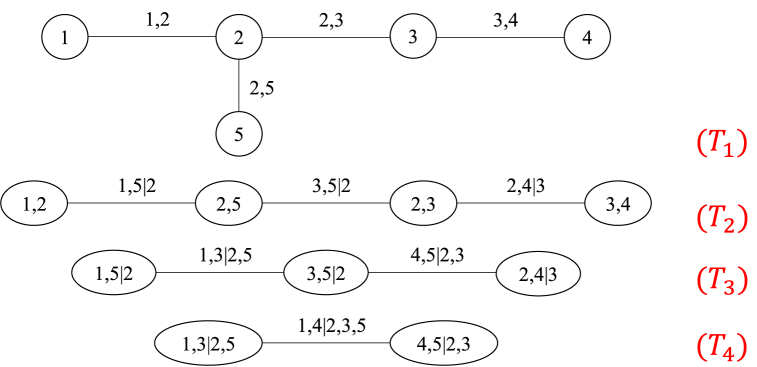
R-Vine structure of 5-dimensional random variables.

For n variables $X_{1},X_{2},...,X_{n}$, the R-Vine Copula structure consists of n−1 trees to form $T=\left(T_{1},T_{2},...,T_{n-1}\right)$, $N_{i}$ and $E_{i}$ represent the vertex set and edge set of the i-th tree, respectively. An example of the R-Vine tree structure with 5 variables is shown in Fig. 1.

A R-Vine of n variables is defined as follows [22]:

Definition 1 $T=\left(T_{1},T_{2},...,T_{n-1}\right)$ is called a R-Vine defined on n variables, if the following conditions are satisfied:

(1) $T_{1}$ is a tree with vertex set $N_{1}=\left\{1,2,...,n\right\}$ and edge set $E_{1}$;
(2) For i=2,...,n−1, $T_{i}$ is a tree with vertex set $N_{i}=E_{i-1}$ and edge set $E_{i}$;
(3) If two vertices in $T_{i}$ are connected by an edge, the edges in $T_{i-1}$ corresponding to the two vertices must have a common vertex.

n-dimensional R-Vine Copula has n−1 trees $T=\left(T_{1},T_{2},...,T_{n-1}\right)$, and these trees have edge sets $E_{1},...,$.

$E_{n-1}$, and the density functions of these n variables are shown as follows [12,23]:

$$f\left(x_{1},...,x_{n}\right)=\prod_{k=1}^{n}f_{k}\left(x_{k}\right)\prod_{i=1}^{n-1}\prod_{e\in E_{i}}c_{v,w|D\left(e\right)}[F\left(x_{v}|x_{D\left(e\right)}\right),$$

(16) $$F\left(x_{w}|x_{D\left(e\right)}\right)]$$

where e={v,w|D(e)} is an edge in $E_{i}$; $c_{v,w|D\left(e\right)}$ is the corresponding Copula density function, where v,w are the two vertices connected by edge e and D(e) is the condition set; $F\left(x_{v}|x_{D\left(e\right)}\right)$ and $F\left(x_{w}|x_{D\left(e\right)}\right)$ are conditional distribution functions.

The conditional distribution function of any random variable x under the condition that the n-dimensional random vector u is known is depicted in Eq. (17) [12] below:

(17) $$F\left(x|u\right)=\frac{\partial C_{x,u_{a}|u_{-a}}\left(F\left(x|u_{-a}\right),F\left(u_{a}|u_{-a}\right);\theta_{x,u_{a}|u_{-a}}\right)}{\partial F\left(u_{a}|u_{-a}\right)}$$

where $u_{a}$ represents a component of n-dimensional random vector u; $u_{-a}$ represents the (n−1)-dimensional vector after removing $u_{a}$ from u; $\theta_{x,u_{a}|u_{-a}}$ is the parameter of Copula function $C_{x,u_{a}|u_{-a}}$.

This paper uses the vineCopula and Copula packages in R language to build the R-Vine Copula model.

## 4 RUL prediction with multi-sensor signals

The RUL of a single degradation signal with a degradation process of X(t) and a threshold of F is L=inf{l:X(t+l)≥F|X(t)<F}, which is proposed in the sense of the first hitting time. Because the engine requires high reliability and high safety, if one sensor signal's degradation amount reaches the threshold, the entire system reaches the threshold. In the online phase, assuming that when the system runs to time $t_{k}$, the observed data of the current time signals is $X_{1:d,k}$, the predicted RUL with multi-sensor degradation signals are depicted in Eq. (18) [24] below:

(18) $$L=\inf\left\{ l_{k}:\begin{array}{c} X_{1}\left(t_{k}+l_{k}\right)\geq F_{1}\\ or\\ \begin{array}{l} \vdots \\ or \end{array}\\ X_{d}\left(t_{k}+l_{k}\right)\geq F_{d} \end{array}\right.|\begin{array}{c} X_{1}\left(t_{k}\right)<F_{1}\\ and\\ \vdots \\ and\\ X_{d}\left(t_{k}\right)<F_{d} \end{array}\}$$

However, it is difficult to obtain the analytical solution of Eq. (16). In this paper, a simulation based method is used to achieve RUL prediction. In the simulation method, the degradation data $X_{1:d,k}=\left(x_{1k},x_{2k},...,x_{dk}\right)$ is known, representing the degradation amount of d degradation signals at the time $t_{k}$; the model parameter $\theta_{1:d}^{M}$ and R-Vine Copula model parameter $\theta^{C}$ of all single signal degradation processes are known, and initialize RUL = 0. After that, the CDF vector $\left(\mu_{1},\mu_{2},...,\mu_{d}\right)$ is obtained by sampling from the R-Vine Copula model. Then use the inverse CDF technique $F_{i}^{-1}\left(\mu_{i},\theta_{i}^{M}\right)$ to obtain the increment of each degradation signal and add it to the current degradation amount of the corresponding degradation signal. As long as the degradation amount of all degradation signals does not reach the threshold, the cycle continues. In order to ensure the reliability and safety of the engine, as long as one degradation signal reaches the threshold, it will exit the cycle and return the results. The simulation based algorithm is shown as follows [24,25].

**Table:** 

Algorithm 1: RUL Prediction		
Data: $\left\{x_{1:d,j}\right\},\left\{\theta^{M},\theta_{1:d}^{C}\right\},\Delta t$		
Result: RUL		
1 RUL=0		
2 $while\;x_{1}<F_{1}\&\ldots \&\;x_{d}<F_{d}\;do$		
3	RUL=RUL+Δt;	
4	$sample\omega_{i}\overset{i.i.d}{˜}Uniform\left(0,1\right),i=1,2,...,d\text{;}$	
5	$\mu_{1}≔\omega_{1}$;	
6	$\mu_{2}≔C_{2|1}^{-1}\left(\omega_{2}|\mu_{1}\right)$;	
7	⋮	
8	$\mu_{d}≔C_{d|d-1}^{-1}\left(\omega_{d}|\mu_{d-1},\ldots ,\mu_{1}\right)$;	
9	fori=1:ddo;	
10	|$x_{i}=x_{i}+F_{i}^{-1}\left(\mu_{i};\theta_{i}^{M}\right)$;	
11	end	
12 end		

where Δt is the increment of observation time, that is, $\Delta t=t_{j}-t_{j-1}$. For the C-MAPSS dataset used in the experiment, all time intervals are equal, that is, Δt=1.

## 5 Experiment

In this section, an aeroengine degradation dataset is employed to demonstrate the effectiveness of the proposed method in this paper and compare the prediction performance with some existed works.

**Fig. 2:**
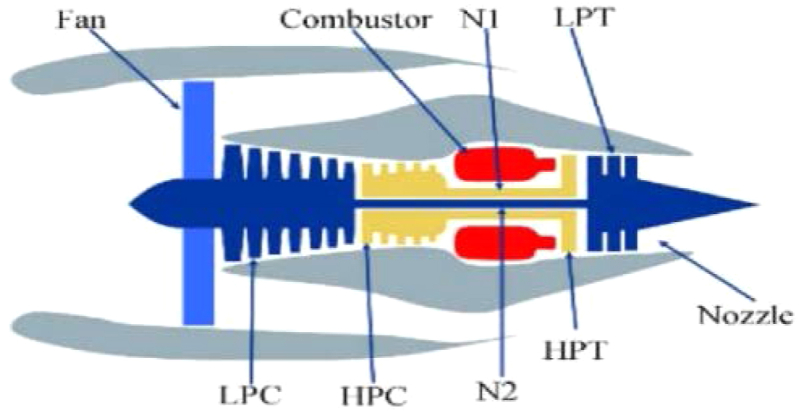
A sketch of main components of the engine.

Experiments use the C-MAPSS dataset provided by NASA [26]. The C-MAPSS dataset is widely used to study the engine degradation trend mechanism. The structural diagram of the turbofan engine is illustrated in Fig. 2.

The training set contains the full life data of multiple engines from normal to failure, and the test set includes many incomplete data that ends before failure, which is used for RUL estimation. Each engine data in the training set or test set consists of 26 multivariate time series, in which the first variable represents the serial number of the engine, the second variable represents the working time in units of cycle (t), the 3rd to 5th variables represent 3 operating parameters (altitude, Mach number, and sea surface temperature) that have a significant impact on engine performance, and the remaining 21 variables represent monitoring data from 21 different sensors. The data set description is shown in Table 1.

**Table 1:** Dataset description of C-MPASS.

Dataset	FD001	FD002	FD003	FD004
Engines for training	100	260	100	249
Engines for testing	100	259	100	248
Operating conditions	1	6	1	6
Fault modes	1	1	2	2

### 5.1 Data preprocessing

The prognostic procedure contains data preprocessing, training, and testing. Since some sensor signals have constant values throughout the operation of the engine, they do not related to the engine degradation process, so they are removed. For degradation data, the order of magnitude is reduced without changing the characteristics of the data, and the mean value of the first ten cyclic data sets is subtracted to facilitate model calculation.

Considering the high similarity of data collected by most sensors [27] in order to reduce the computational complexity of the prediction model, 6 representative sensor data were selected for experiments. Since the FD001 and FD003 data sets are collected under the same working conditions, the sensor data numbered 10 is not unnecessary. The selected sensor is shown in Table 2.

**Table 2:** Detailed description of 6 sensors.

	Symbol	Description	Units
3	T_30_	Total temperature at HPC outlet	K
4	T_50_	Total temperature at LPT outlet	K
7	P_30_	Total pressure at HPC outlet	kPa
10	e_pr_	Engine pressure ratio	
11	Ps_30_	Static pressure at HPC outlet	Pa
12	phi	Ratio of fuel flow to Ps30	m^3^(Pa∙s)

**Fig. 3:**
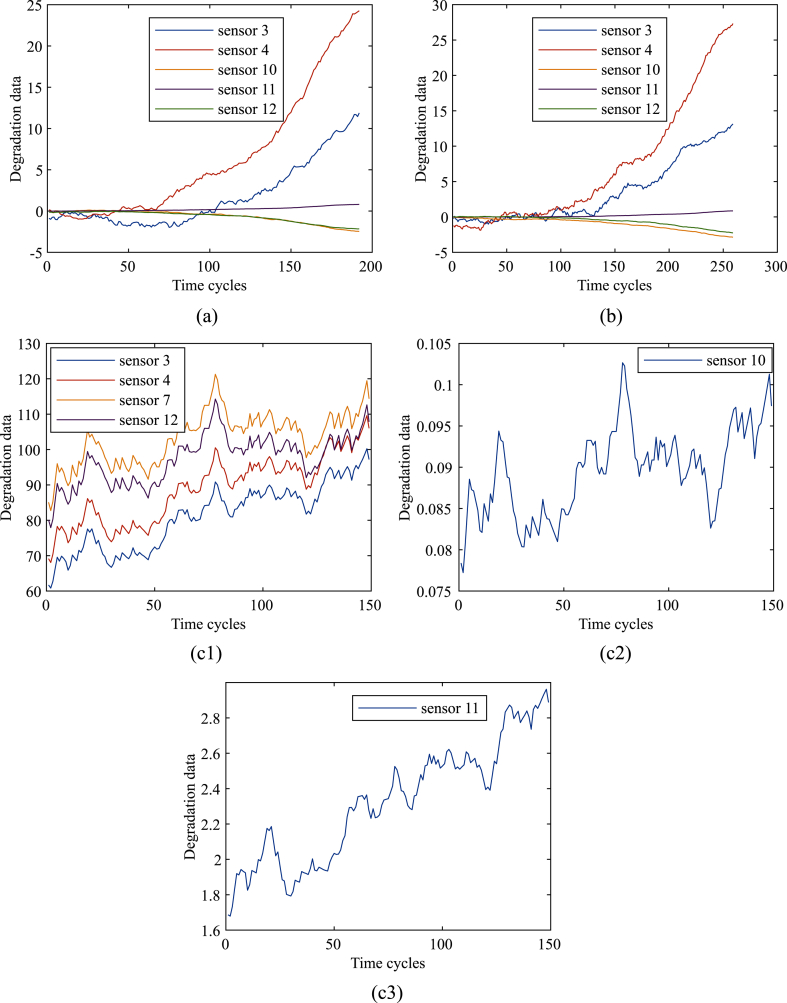

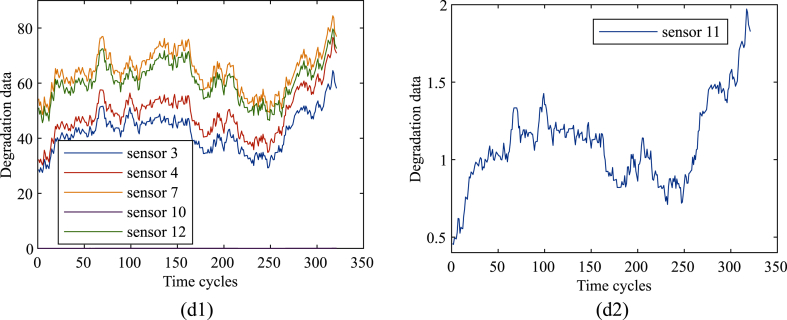
Observation data of the sensor after preprocessing, (a) Smoothed FD001 dataset; (b) Smoothed FD003 dataset; (c) Smoothed FD002 dataset: (c-1) Smoothed results of sensors 3, 4, 7 and 12. (c-2) Smoothed result of sensor 10. (c-3) Smoothed result of sensor 11; (d) Smoothed FD004 dataset: (d-1) Smoothed results of sensors 3, 4, 7, 10 and 12. (d-2) Smoothed result of sensor 11.

Because of the noise and large random fluctuations, the experiment began to filter and smooth the data. Taking the No.1 sensor data of the first engine as an example, FD001, FD002, FD003 and FD004 datasets are processed as shown in Fig. 3 a-d.

### 5.2 RUL prediction of single degradation signal

This article uses a nonlinear Wiener to model the engine degradation process, and the drift coefficient $\mu \left(t,\beta \right)=\beta t^{\beta -1}$ as the index function. Based on Eq. (12), the optimal solution is obtained by fminsearch function of MATLAB, the prior values of $\sigma_{a,0}^{2}$, $\sigma_{b,0}^{2}$ and β are obtained. Then prior value of $\mu_{a,0}$ can be obtained from Eq. (11). The prior parameters of subsets FD001, FD002, FD003 and FD004 are shown in Table 3.

**Table 3:** Parameters estimation results.

	FD001				FD002			
	$\mu_{a,0}$	$\sigma_{a,0}^{2}$	$\sigma_{b,0}^{2}$	β	$\mu_{a,0}$	$\sigma_{a,0}^{2}$	$\sigma_{b,0}^{2}$	β
3	2.8623 × 10^−4^	2.8984 × 10^−18^	0.2703	1.9948	0.0111	2.2599 × 10^−10^	3.6531	1.6454
4	5.631 × 10^−4^	3.0739 × 10^−18^	0.2795	2.0490	0.2515	4.7119 × 10^−10^	6.5387	1.1312
7	1.112 × 10^−3^	1.1944 × 10^−10^	0.09	1.3667	0.5943	6.7519 × 10^−10^	5.4541	1.0527
10	–	–	–	–	0.1078	2.1169 × 10^−10^	3.9486	1.3123
11	1.6548 × 10^−4^	2.5422 × 10^−20^	0.3404	1.6224	0.0268	6.8284 × 10^−12^	2.8430	1.1109
12	0.0097	1.115 × 10^−4^	0.515	1.667	0.5697	3.1673 × 10^−12^	2.9377	1.0327

	FD003				FD004			
	$\mu_{a,0}$	$\sigma_{a,0}^{2}$	$\sigma_{b,0}^{2}$	β	$\mu_{a,0}$	$\sigma_{a,0}^{2}$	$\sigma_{b,0}^{2}$	β
3	8.4875 × 10^−4^	1.91 × 10^−16^	1.1485	1.7411	0.0112	1.009 × 10^−10^	5.5571	1.5654
4	0.181	4.9076 × 10^−16^	1.6045	0.9295	0.6531	5.2696 × 10^−6^	6.5387	1.001
7	1.1537 × 10^−6^	1.0005 × 10^−20^	2.9721	2.8111	0.6277	1.3884 × 10^−10^	3.6956	1.0301
10	–	–	–	–	0.001091	1.4787 × 10^−7^	4.9218	1.701
11	3.2131 × 10^−4^	1.7593 × 10^−20^	2.9112	1.8515	0.0244	1.619 × 10^−10^	9.5386	1.2108
12	1.5799 × 10^−6^	1.1677 × 10^−22^	2.1140	2.7296	1.1562	3.070 × 10^−10^	1.9384	1.0101

**Fig. 4:**
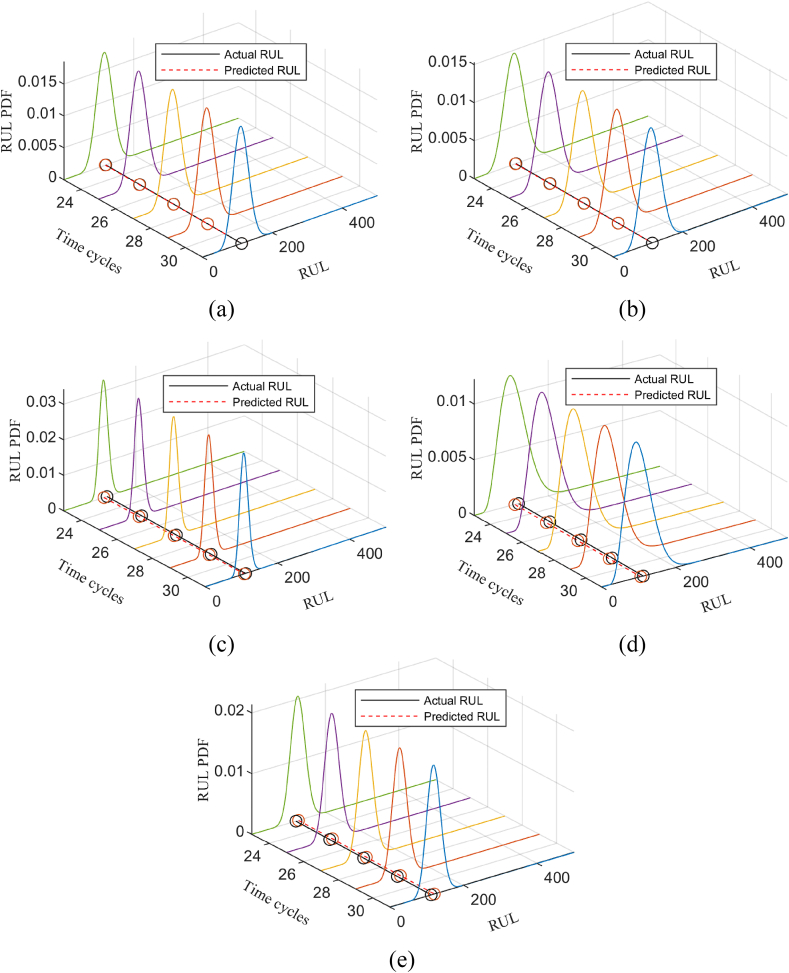
PDF and RUL, (a) PDF and RUL of sensor 3; (b) PDF and RUL of sensor 4; (c) PDF and RUL of sensor 7; (d) PDF and RUL of sensor 11; (e) PDF and RUL of sensor 12.

Select the last five times of the test set (the time interval is 1) for RUL prediction. The PDF and RUL of the last five times of all sensors are shown in Fig. 4 a-e, also taking the No.1 engine as an example.

### 5.3 RUL prediction of multi-sensor signals based on R-Vine copula

**Fig. 5:**
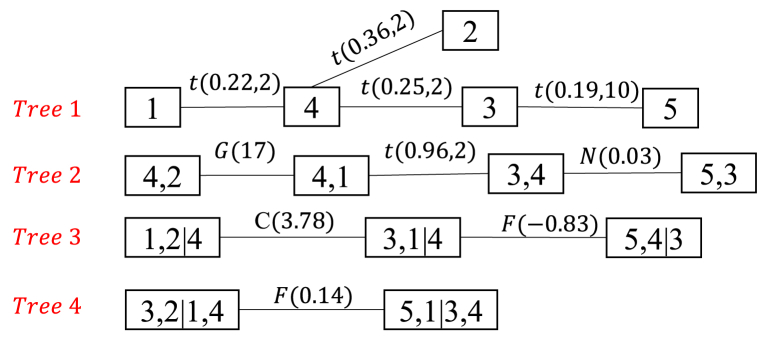
R-Vine structure of sensors.

The RUL prediction based on R-Vine Copula is calculated according to the algorithm 1 in Section 4. Also taking FD001 as an example, the R-Vine Copula model node pair Copula function types and parameters to be solved are listed in Table 4, and the R-Vine tree structure is shown in Fig. 5. The numbers 1, 2, 3, 4, and 5 represent Sensors 3, 4, 7, 11, 12.

**Table 4:** Function selection and parameter estimation at each node of R-Vine Copula mode.

Tree level	variable	optimal Copula	
		Type	Parameters estimation
Tree 1	4,2	Student t copula	(0.36,2)
	4,1	Student t copula	(0.22,2)
	3,4	Student t copula	(0.25,2)
	5,3	Student t copula	(0.19,10)
Tree 2	1,2; 4	Gumbel copula	17.00
	3,1; 4	Student t copula	0.96
	5,4; 3	Gaussian copula	0.03
Tree 3	3,2; 1,4	Clayton copula	3.78
	5,1; 3,4	Frank copula	−0.83
Tree 4	5,2; 3,1,4	Frank copula	0.14

**Fig. 6:**
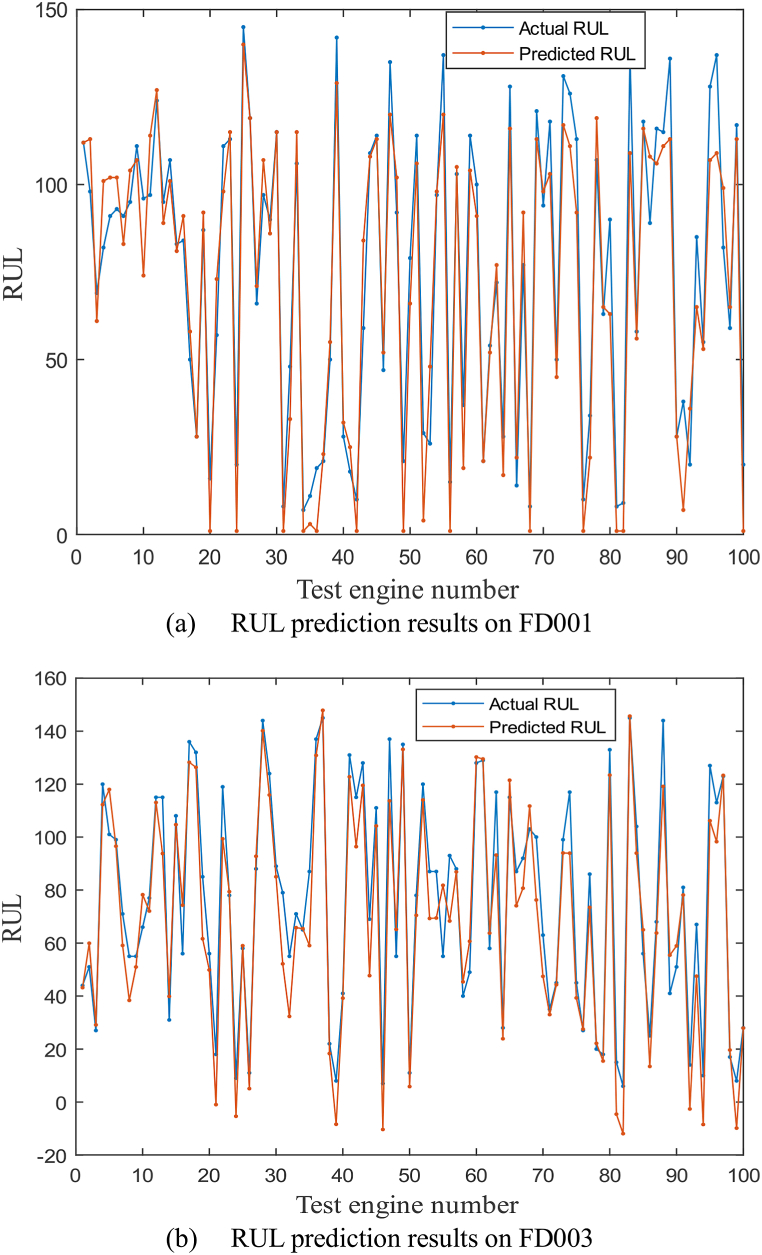
RUL prediction for multi-sensor degradation signals, (a) RUL prediction results on FD001; (b) RUL prediction results on FD003.

The RUL at the last moment of all engines in test set is calculated and compared with the real RUL. Taking FD001 and FD003 as examples, compare the predicted RUL and actual RUL of 100 engines. The result is shown in Fig. 6 a, b.

### 5.4 Comparisons with other methods

In this section, compare the method proposed in this paper with other methods. The error between predicted RUL and actual RUL characterizes the prediction performance of the model. The root means square error (RMSE) and score function are adopted as statistical indices to assess the performance of the proposed model, which can be depicted in Eqs. (19), (20), (21) below:

(19) $$d_{i}=y_{i}-{\hat{y}}_{i}$$

(20) $$RMSE=\sqrt{\frac{1}{n}\sum_{i=1}^{n}{\left(d_{i}\right)}^{2}}$$

(21) $$Score=\left\{ \begin{array}{c} \sum_{i=1}^{n}e^{-\frac{d_{i}}{13}}-1,d_{i}<0\\ \sum_{i=1}^{n}e^{\frac{d_{i}}{10}}-1,d_{i}\geq 0 \end{array}\right.$$

where n is the number of testing engine units and $d_{i}$ means predicted RUL ${\hat{y}}_{i}$ minus real RUL $y_{i}$. It can be seen from the score indicator that it is biased toward the forward prediction and punishes the lagging forecast. Therefore, the lower the score index, the more reliable the model is.

Table 5 compares the method proposed in this paper with other methods [[28], [29], [30], [31], [32]]. For datasets FD001 and FD003, the method proposed in this paper performs better on score indicator.

**Table 5:** Comparison of method in this study and other methods.

Methods	RMSE				Score			
	FD001	FD002	FD003	FD004	FD001	FD002	FD003	FD004
CNN [28]	18.45	30.29	19.82	29.16	1286.70	13570.00	1596.20	7886.40
GB [29]	15.67	29.09	16.84	29.01	474.01	87280.06	576.72	17817.92
RF [29]	17.91	29.59	20.27	31.12	479.75	70456.86	711.13	46567.63
DBN [29]	15.21	27.12	14.71	29.88	417.59	9031.64	442.43	7954.51
CNN + FNN [30]	12.61	22.36	12.64	22.43	274	10412	284	12466
Auto-Encoder [31]	13.58	19.59	19.16	22.15	220	2650	1727	2901
BiGRU-TSAM [32]	12.56	18.94	12.45	20.47	213.35	2264.13	232.86	3610.34
The proposed method	12.81	19.56	13.32	24.14	**197.23**	2303.19	**194.22**	4056.87

## 6 Conclusion

RUL prediction with multi-sensor degradation signals is a more realistic and challenging issue than the cases of a single degradation signal. This work investigated a novel aeroengine RUL prediction method with multi-sensor data.

1) The correlation between different degradation signals and the engine degradation process is different, so using multi-sensor degradation signals to predict engine RUL is appropriate. The RUL prediction model proposed in this paper can make full use of the correlation between different degradation signals.
2) The degradation process of the engine is nonlinear, and the Wiener process cannot accurately describe the degradation process of the engine with nonlinear characteristics, so this paper uses the nonlinear Wiener process to model the degradation process of the engine.
3) The experimental results verify the effectiveness of the proposed method and compare it with other methods. For engines with different degradation processes, the nonlinear Wiener process can well describe engine process with obvious degradation trend, such as the FD001 and FD003 subsets. The proposed method has higher accuracy in the score index, and the forward prediction can make the aircraft safer.

Although the proposed approach can effectively describe the degradation process with multi-sensor data, there are still some problems that need to be investigated in the future. Because of the different operating conditions and fault modes, the proposed method does not perform very well on the subsets FD002 and FD004. In such cases, deep learning models are often a better choice.

In the future, we would like to explore further novel methods to tackle other systems' remaining useful life prediction problems with multi-sensor data.

## Declaration of competing interest

The authors declare that they have no known competing financial interests or personal relationships that could have appeared to influence the work reported in this paper.

## References

[1] Ren Z., Si X., Hu C. (2019). Remaining useful life prediction method for engine combining multi-sensors data[J]. Acta Aeronautica Astronautica Sinica.

[2] Xue X., Tian J., He S. (2021). Nonlinear degradation assessment of aircraft components monitored by multi-sensors[J]. Acta Aeronautica Astronautica Sinica.

[3] Caesarendra W., Widodo A., Thom P.H. (2011). Combined proba-bility approach and indirect data-driven method for bearing degradation prognostics[J]. IEEE Trans. Reliab..

[4] Gebraeel N.Z., Lawley M.A., Li R. (2005). Residual-life distributions from component degradation signals: a Bayesian approach[J]. IIE Trans..

[5] Saxena A., Goebel K., Simon D. (2008).

[6] Si X., Wang W., Hu C. (2012). Remaining useful life estimation based on a nonlinear diffusion degradation process[J]. IEEE Trans. Reliab..

[7] Si X., Hu C., Zhou D. (2013). Nonlinear degradation process modeling and remaining useful life estimation subject to measurement error[J]. Acta Autom. Sin..

[8] Liu Y., Xiao Q., Yang G. (2021). Failure probability analysis of service long-span bridge girder based on optimal R-Vine Gaussian copula model[J]. Journal Of Tong Ji University (Nature Science).

[9] Zhang J., Jiang Y., Li X. (2022). An adaptive remaining useful life prediction approach for single battery with unlabeled small sample data and parameter uncertainty[J]. Reliab. Eng. Syst. Saf..

[10] Zhang J., Tian J., Li M. (2022). A parallel hybrid neural network with integration of spatial and temporal features for remaining useful life prediction in prognostics[J]. IEEE Trans. Instrum. Meas..

[11] Zhang J., Li X., Tian J. (2023).

[12] Zhang J., Li X., Tian J. (2023). A variational local weighted deep sub-domain adaptation network for remaining useful life prediction facing cross-domain condition[J]. Reliab. Eng. Syst. Saf..

[13] Wu W., Wang K., Han B. (2015). A versatile probability model of photovoltaic generation using pair copula construction[J]. IEEE Trans. Sustain. Energy.

[14] Si X., Hu C., Li J. (2015). Remaining useful life prediction of nonlinear stochastic degradation systems subject to uncertain measurement[J]. J. Shanghai Jiaot. Univ..

[15] Dong G., Chen Z., Wei j (2018). Battery health prognosis using Brownian motion modeling and particle filtering[J]. IEEE Trans. Ind. Electron..

[16] Liu K., Gebraeel N., Shi J. (2013). A data-level fusion model for developing composite health indices for degradation modeling and prognostic analysis[J]. IEEE Trans. Autom. Sci. Eng..

[17] Si X.S., Wang W., Hu C. (2012). Remaining useful life estimation based on a nonlinear diffusion degradation process[J]. IEEE Trans. Reliab..

[18] Si X., Hu C. (2005).

[19] Si X., Wang W., Chen M. (2013). A degradation path-dependent approach for remaining useful life estimation with an exact and closed-form solution[J]. Eur. J. Oper. Res..

[20] Zeng W., Xu M., Song S. (2022). Joint probability distribution and risk identification of extreme precipitation based on R-Vine copula function[J]. Water Resources Protection.

[21] Sklar M. (1959). Fonctions de repartition an dimensions et leurs marges. J]. Publ. inst. statist. univ. Paris.

[22] Guo R. (2018).

[23] Dissmann J., Brechmann E., Czado C. (2013). Selecting and estimating regular vine copula and application to financial returns[J]. Comput. Stat. Data Anal..

[24] Fang G., Pan R. (2021). On multivariate copula modeling of dependent degradation processes[J]. Comput. Ind. Eng..

[25] Czado C. (2019).

[26] Saxena A., Kai G., Simon D. (2008).

[27] Chao M.A., Kulkarni C., Goebel K. (2022). Fusing physics-based and deep learning models for prognostics[J]. Reliab. Eng. Syst. Saf..

[28] Sateesh B., Zhao P., Li X. (2016).

[29] Zhang C., Lim P., Qin A. (2016). Multiobjective deep belief networks ensemble for remaining useful life estimation in prognostics[J]. IEEE Transact. Neural Networks Learn. Syst..

[30] Li X., Ding Q., Sun J.Q. (2018). Remaining useful life estimation in prognostics using deep convolution neural networks[J]. Reliab. Eng. Syst. Saf..

[31] Yu W., Kim I., Mechefske C. (2020). An improved similarity-based prognostic algorithm for RUL estimation using an RNN autoencoder scheme[J]. Reliab. Eng. Syst. Saf..

[32] Zhang J., Jiang Y., Wu S. (2022). Prediction of remaining useful life based on bidirectional gated recurrent unit with temporal self-attention mechanism[J]. Reliab. Eng. Syst. Saf..

